# Migration and Accumulation of Octachlorodipropyl Ether in Soil-Tea Systems in Young and Old Tea Gardens

**DOI:** 10.3390/ijerph14091033

**Published:** 2017-09-08

**Authors:** Min Liao, Yan-Hong Shi, Hai-Qun Cao, Qing-Kui Fang, Jin-Jing Xiao, Ri-Mao Hua

**Affiliations:** 1 School of Plant Protection, Anhui Agricultural University, Hefei 230036, China; liaomin3119@126.com (M.L.); qkfang@163.com (Q.-K.F.); xiaojj187012@163.com (J.-J.X.); 2 Provincial Key Laboratory for Agri-Food Safety, Anhui Agricultural University, Hefei 230036, China; shiyh@ahau.edu.cn (Y.-H.S.); rimaohua@126.com (R.-M.H.); 3 School of Resource & Environment, Anhui Agricultural University, Hefei 230036, China

**Keywords:** octachlorodipropyl ether, soil-tea system, fresh tea leaf, migration, accumulation

## Abstract

The migration and accumulation of octachlorodipropyl ether (OCDPE) in soil-tea systems were investigated using a gas chromatography-electron capture detector (GC-ECD) method in young and old tea gardens. When the residual concentration of OCDPE was 100 g a.i. hm^−2^ in soils, the peak concentrations of OCDPE in fresh leaves of young and old tea plants were 0.365 mg/kg and 0.144 mg/kg, taking 45 days and 55 days, respectively. Equations for the accumulation curves of OCDPE in fresh leaves of young and old tea plants were C_t_ = 0.0227e^0.0566t^ (R^2^ = 0.9154) and C_t_ = 0.0298e^−0.0306t^ (R^2^ = 0.7156), and were C_t_ = 3.8435e^0.055t^ (R^2^ = 0.9698) and C_t_ = 1.5627e^−0.048t^ (R^2^ = 0.9634) for dissipation curves, with a half-life of 14.4 days and 12.6 days, respectively. These results have practical guiding significance for controlling tea food safety.

## 1. Introduction

Obtained from the tender leaves of the plant *Camellia siensis* (L.), tea is one of the oldest and most popular beverages in the world, especially in Asia [1,2]. Tea is rich in phenolics such as catechins and flavonols, and is important in antimutagenic and anticlastogenic treatments because of its strong antioxidant activities [3,4]. It also contains minerals and vitamins, which may increase its antioxidant potential. Similar to other agriculture commodities, tea is prone to attack by various pests and diseases. To ensure its quality and safety, pyrethroid, organophosphorus, and carbamate insecticides have been widely used in nearly every period of its cultivation, storage, and product manufacturing processes [3,5]. 

**Figure 1:**
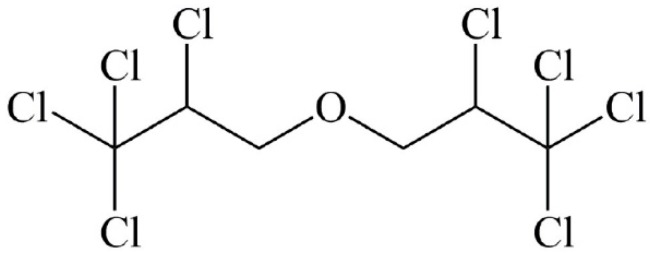
Structure of octachlorodipropyl ether (OCDPE).

Octachlorodipropyl ether (OCDPE) (Figure 1) is a chloroalkyl ether with insecticidal and synergistic activity [6,7]. It has been applied as an insecticide synergist for pyrethroid, organophosphorus, and carbamate insecticides, which are widely used in commercial agriculture [8,9]. The maximum residue limits (MRL) of OCDPE in tea were established by the European Union (EU) in 2003 [10], and it has been banned in China because of its subacute or chronic toxicity such as subacute hepatotoxicity, cytotoxicity, carcinogenicity, and contact allergenicity [11,12]. However, OCDPE residues still could be detected in agricultural products and environmental samples. OCDPE was still widely used in household insecticides as synergist; thus, it was suggested that the main source of OCDPE in tea maybe the absorption from the combustion of mosquito coils and the use of aerosols [13,14]. Therefore, it is necessary to study the OCDPE residues in soil-plant systems to guarantee human and environmental safety.

OCDPE residues in tea and soil in tea gardens have been reported. For instance, the dissipation behavior of OCDPE residues was evaluated during tea planting [15,16], residual dynamics of OCDPE were analyzed in tea garden soils [15,17], OCDPE residues were determined in tea by gas-chromatography [13,18,19], and dietary risk of OCDPE residues was assessed during brewing processes for tea [15]. OCDPE accumulates in soils from wastewater irrigation and aerial deposition, and then plants uptake OCDPE from soils through the roots [20,21,22]. However, few reports were concentrated on the migration and accumulation of OCDPE in soil-tea systems in tea gardens.

The aim of this research is to analyze the migration and accumulation of OCDPE in soil-tea systems in young and old tea gardens. Here, we prepared 10% OCDPE emulsifiable concentrate, established a method for the determination of OCDPE residue in fresh tea leaves using GC-ECD, and investigated the migration and accumulation of OCDPE in soil-tea systems. These results showed the migration and accumulation of OCDPE in soil-tea systems, which can serve as a guide to control the food safety of tea and other agricultural products.

## 2. Materials and Methods 

### 2.1. Chemicals and Instruments

Octachlorodipropyl ether (OCDPE, 99.6%) was purchased from National Pesticide Product Quality Supervision and Inspection Center (Beijing, China). Anhydrous sodium sulfate, ethyl acetate, *n*-hexane, petroleum ether, and xylene (analytical grade) were all purchased from Sinopharm Group Chemical Reagent Co., Ltd. (Shanghai, China). Agricultural emulsifier 2201 (Calcium dodecylbenzenesulfonate: styrene, phenol, formaldehyde condensates, polyoxyethylene ether (emulsifier 400-3#): dimethylbenzene = 30:43:26, *v/v*) was purchased from Haian Petrochemical Factory (Nantong, China).

Gas chromatography (GC) was performed using an Agilent 6890 Series instrument from Agilent Technologies (Santa Clara, CA, USA). An EYELAN-1100 rotary evaporator was used from the Shanghai Ailang Instruments Co., Ltd. (Shanghai, China). A TG16-WS high-speed centrifuge was used from Changsha Xiangyi Centrifuge Instrument Co., Ltd. (Changsha, China). A SB-3200 ultrasonic cleaner was used from Kunshan Ultrasonic Instrument Co., Ltd. (Shanghai, China). A DHG-9070A electrothermal oven thermostat blast was used from the Shanghai Yiheng Science and Technology Co., Ltd. (Shanghai, China).

### 2.2. Preparation of 10% OCDPE Emulsifiable Concentrate (EC)

To prepare 10% OCDPE EC, 5.00 g agricultural emulsifier 2201 (emulsifier) was added to 5.40 g OCDPE crude oil (purity: 93%) in a 150 mL beaker, and then xylene (solvent) was added to the total weight of 50.00 g. Having been stirred with a glass bar and mixed well, 10% OCDPE EC was transferred into a brown bottle and kept at room temperature away from light. The quality of the preparation meets the standard.

### 2.3. Migration and Accumulation Experiment of OCDPE in Soil-Tea Systems

Young (7 years old) and old (15 years old) tea gardens were chosen for soil application treatments (no pesticides were used in the test area). At about 30 cm away from the main stem of tea trees, a dosing ditch was dug with a depth of about 20 cm, as the root biomass of tea is mainly distributed in 0~20 cm of soil layer, accounting for more than 90% of the total. A certain amount of 10% OCDPE EC and fine sand (passed through a 20 mesh screen) mix was evenly applied in the ditch, and thus the treatment of OCDPE 100.00 g active ingredient per square hectometer (a.i. hm^−2^) was applied. Each plot was 30 m^2^, and the treatment was repeated three times.

Soil and tea fresh leaf samples were collected at 1, 3, 5, 7 and 10 days after the treatment, respectively. After 10 days, soil and fresh leaf samples were collected every five days until no OCDPE was detected in soil and fresh leaf samples. The soil samples were conducted by sampling in five locations for each subsequent testing. Furthermore, 0–10 cm of dosing ditch soils were collected and then mixed evenly, air dried, and crushed over a 40 mesh sieve. For the fresh tea leaves, a bud with a leaf was removed from the plant, deionized by washing with water, dried, and then ground using a food mixer before being measured.

### 2.4. Sample Extraction and Clean-Up

#### 2.4.1. Fresh Tea Leaf Sample Treatment

Fresh tea leaf samples (20.00 g) were extracted with 80 mL of acetone/n-hexane (2:1, *v/v*), mixed solution in a 250 mL triangle bottle, and thoroughly shaken on the rotary shaker for 1 h. After being passed through a Buchner funnel vacuum filter, the filter residue was washed with acetone (10 mL × 3), the filtrate was transferred to a 500 mL separatory funnel, and the anhydrous sodium sulfate was added. Following extraction with n-hexane (20 mL × 3), the organic phase passed through the small funnel of anhydrous sodium sulfate, and was concentrated close to dryness at 40 °C.

A Florisil solid phase extraction (SPE) was pre-leached by 5.00 mL of n-hexane. The fresh tea leaf extract was washed with n-hexane (1 mL × 2), and transferred to the SPE column which was rinsed by 10.00 mL acetone:*n*-hexane (1/9, *v/v*) mixed solution. Then, the eluate was collected and concentrated to dryness at 40 °C. The volume was set to 5.00 mL in a volumetric flask with *n*-hexane; the extract was filtered through an Acrodisc^®^ Syringe Filter (0.2 mm Supor^®^ Membrane, Pall Corporation, Port Washington, NY, USA), and then the filtrate was measured.

#### 2.4.2. Soil Sample Treatment

In our previous study, soil sample treatment and the spiked experiment were reported [17]. OCDPE was added to the untreated control samples of soil at 0.01, 0.1, and 1.0 mg/kg. The recoveries of OCDPE ranged from 82.3% to 101.5%, and the relative standard deviations (RSDs) were between 7.4% and 9.9% for the soil samples.

### 2.5. Sample Extraction and Clean-Up

This proposed method was performed on a GC coupled with electron capture detector (ECD). A 30 m × 0.25 mm × 0.25 μm HP-5 capillary column (Supelco Inc., Bellefonte, PA, USA) was used. The temperature of the injector operated in splitless mode (volume injected 2 μL) was held at 260 °C. The oven temperature program ran from 150 °C (held for 1 min) to 170 °C (held for 3 min) at a rate of 4 °C/min, and then to 190 °C (held for 4 min) at a rate of 5 °C/min; the detector temperature was 320 °C. The carrier gas was N_2_ (purity > 99.999%) and the flow rate was 1.0 mL/min.

### 2.6. Statistical Analysis

Data are expressed as the means ± standard deviation (S.D.). All figures were drawn using the software Origin Pro 9.0 (Origin Lab Corporation, Northampton, MA, USA). The relationship between OCDPE residues and time is C_t_ = C_0_e^−kt^ [23], where C_t_ (mg/kg) is the residue after time t, C_0_ (mg/kg) is the initial residue, and k is the dissipation rate (accumulation rate) constant (d^−1^). The exponential equation was analyzed using the Exp2PMod1 function of the software Origin Pro 9.0.

## 3. Results and Discussion

### 3.1. Recovery Study

The limits of detection (LOD) were determined as the sample concentration of OCDPE at a signal-to-noise ratio of 3:1 by GC-ECD. The LOD of OCDPE was estimated at 0.005 mg/kg for the soil and fresh tea leaf samples. The analyte recoveries for the spiked samples are summarized in Table 1. OCDPE was added to the untreated control samples at 0.01, 0.05, and 0.5 mg/kg for the fresh tea leaves. For method validation, the control and treated samples were analyzed under the same conditions. We repeated the measurement five times at each spiked level. The recoveries of OCDPE ranged from 77.7% to 92.3%, and the RSDs were between 8.6% and 10.4% for the fresh tea leaf samples. Thus, these results demonstrated that the GC-ECD method could be used for tea sample determination.

### 3.2. Migration and Accumulation of the OCDPE in Soil-Tea Systems

**Figure 2:**
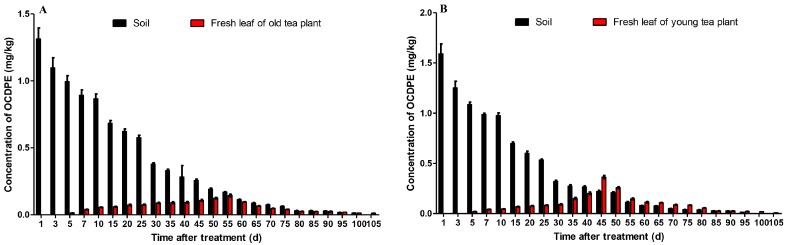
Migration and accumulation of OCDPE in soil-tea systems (n = 3). (**A**) Fresh leaf samples of old tea plants; (**B**) fresh leaf samples of young tea plants.

The data on the migration and accumulation of OCDPE obtained in the soil-tea systems are shown in Figure 2. A gradual and continuous deterioration of OCDPE in soil samples was observed (Figure 2A,B). In contrast, the concentration of the OCDPE in the fresh tea leaves increased gradually until reaching a peak (Figure 2A,B), where the OCDPE concentrations in the fresh leaves of young and old tea plants were 0.365 mg/kg and 0.144 mg/kg, taking 45 days and 55 days, respectively. Similar degradation trends were also observed between young and old tea plants, and their initial residues were 1.3130 and 1.5910 mg/kg, respectively. Compared with spraying, the sand-mixed method required a longer time for the pesticide to accumulate in the soil [24], which led to a significant reduction after 2 days.

The following are factors affecting the pesticide’s persistence in crops: environmental factors such as temperature and pH, physical and chemical properties of the pesticide (e.g., water solubility, stability, and volatility), and crop characteristics [25,26]. From the above investigation, it is proposed that the growth dilution factor might have played a basic role in the migration and accumulation of OCDPE. Further, OCDPE is fairly persistent in the crop and the environment, and this warrants further study.

### 3.3. Accumulation and Dissipation of OCDPE in Fresh Leaves of Tea Plants

**Figure 3:**
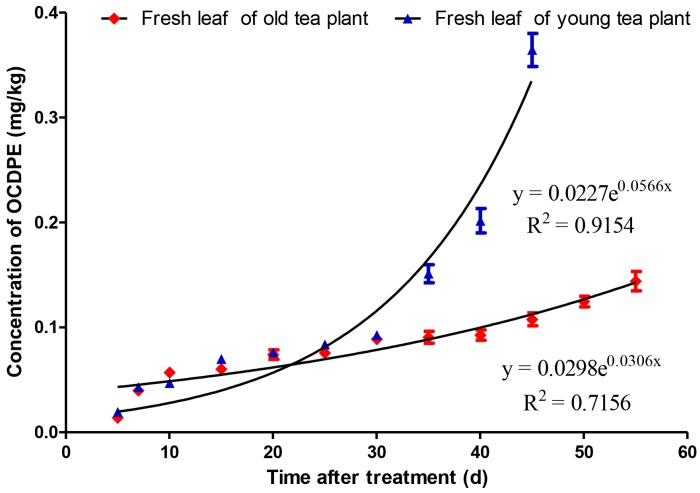
The accumulation curve of OCDPE in the fresh leaves of young and old tea plants (n = 3).

Tea plants absorb OCDPE from the soils through the roots, after which it migrates into fresh leaves. The OCDPE concentration in the fresh leaves of tea plants increased gradually until a peak was reached (Figure 3). The time to reach the peak of the OCDPE concentration in the fresh leaves of young tea plants ranged from 5 to 45 days, and the dynamics could be described by the equation C_t_ = 0.0227e^0.0566t^ (R^2^ = 0.9154). The time to reach the peak of the OCDPE concentration in the fresh leaves of old tea plants ranged from 5 to 55 days, and the dynamics could be described by the equation C_t_ = 0.0298e^−0.0306t^ (R^2^ = 0.7156). 

**Figure 4:**
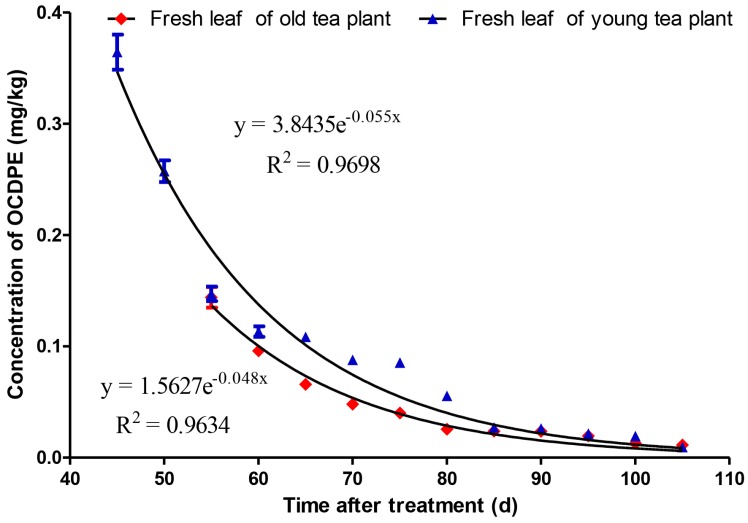
The dissipation curve of OCDPE in the fresh leaves of young and old tea plants (n = 3).

When the OCDPE concentration in the fresh leaves of tea plant reached the peak, a gradual and continuous dissipation of OCDPE in the fresh leaf tea plant samples was observed (Figure 4). The half-life values (T_1/2_) for the degradation of OCDPE in the fresh leaves of young and old tea plants were calculated to be 12.6 days and 14.4 days, respectively, and the dynamics could be described by the equations C_t_ = 3.8435e^0.055t^ (R^2^ = 0.9698) and C_t_ = 1.5627e^−0.048t^ (R^2^ = 0.9634). Therefore, compared to old tea plants, the accumulation rate and dissipation rate of OCDPE in young tea plants were faster, and the accumulation amount of OCDPE in young tea plants was higher. The tea in different growth periods has different nutrient requirements, and a stronger absorption and metabolism occur in the perinatal period, leading to young tea plants have a higher enrichment capacity. As Zong et al. (2007) reported, the heavy metal (Pb) content of young leaves in immature tea plants was higher than that in mature plants, and the distribution arrangement of Pb content was higher in the roots in old tea plants [27]. In this study, a higher enrichment in young tea, which was consistent with that found in earlier work, demonstrated that growth period is a key factor affecting the accumulation and dissipation of OCDPE.

## 4. Conclusions

In summary, we developed a GC-ECD method for the detection of OCDPE residues, and successfully investigated the migration and accumulation of OCDPE in soil-tea systems. Based on the results, tea plants can uptake OCDPE from contaminated soils. OCDPE concentration in the fresh leaves of tea plants increased gradually until reaching a peak, and then gradual and continuous deterioration was observed. By comparing the accumulation rate, accumulation amount, and dissipation rate of the fresh leaves of young and old tea plants, the results demonstrated that the absorption and metabolism of young tea plants were stronger than those of old tea plants. 

## Figures and Tables

**Table 1:** Recovery studies of samples spiked with OCDPE by GC-ECD (n = 3).

Sample	Spiked Level (mg/kg)	Recovery (%)	RSD ^a^ (%)
fresh tea leaf	0.01	92.3 ± 6.51	10.2
	0.05	84.3 ± 5.84	10.4
	0.5	77.7 ± 4.67	8.6

^a^ RSD: relative standard deviation; OCDPE: octachlorodipropyl ether; GC-ECD: Gas Chromatography-Electron Capture Detector.
